# Bile Microbiome in Patients with Recurrent Common Bile Duct Stones and Correlation with the Duodenal Microbiome

**DOI:** 10.3390/life12101540

**Published:** 2022-10-03

**Authors:** Jungnam Lee, Jin-Seok Park, Jaewoong Bae, Sohee Lee, Yeonju Hwang

**Affiliations:** 1 Department of Internal Medicine, Inha University Hospital, Inha University School of Medicine, Incheon 22332, Korea; 2 Research and Development Institute, BioEleven Co., Ltd., Seoul 06220, Korea

**Keywords:** common bile duct stone recurrence, microbiome, enterococcus

## Abstract

Background: Common bile duct (CBD) stone recurrence is a common late adverse event after CBD stone treatment. In this preliminary study, we compared the bile fluid and duodenum microbial profiles of patients with or without recurrent CBD stones to identify risk factors associated with recurrence. Methods: Bile fluid samples of 47 consecutive patients who underwent ERCP for biliary diseases were subjected to microbiome analysis. Nineteen patients were stone-recurrent (SR), and 28 patients were non-stone-recurrent (NSR). Paired samples (duodenum biopsy tissue and bile fluid samples) from five SR patients were used to compare microbiome compositions in the biliary system and duodenum. In addition, we compared the microbiome compositions of these duodenal tissue samples with those 12 controls (gastric ulcer patients without recurrent CBD stones). Results: Enterococcaceae_unclassified and enterococcus were more abundant in bile fluid in the SR group than in the NSR group (*p* = 0.002 and *p* = 0.003, respectively). A comparison of the microbiome compositions of duodenum tissue and bile fluid samples of the five recurrent CBD stone patients revealed proteobacteria compositions were almost identical from the phylum to genus level. In these five patients, alpha and beta diversities were no different in bile fluid and duodenal tissues. Furthermore, a comparison of the microbiome compositions of duodenal mucosa in patients with recurrent CBD stone patients (*n* = 5) and controls (*n* = 12) revealed significant differences between microbiome compositions. Conclusions: Enterococcus seems to contribute to CBD stone development. Furthermore, our results indicate that retrograde migration of the duodenal microbiome may contribute to bile microbiome alterations. We recommend that more research be conducted to confirm this hypothesis.

## 1. Introduction

Recurrent common bile duct (CBD) stones are defined as CBD stones that recur more than 6 months after complete clearance [1]. Guidelines recommend endoscopic retrograde cholangiopancreatography (ERCP) with endoscopic sphincterotomy (EST) as a first-line treatment for CBD stones [2,3]. Nonetheless, studies have reported recurrence rates of 4 to 24% after ERCP with EST [4,5]. CBD stone recurrence is an important health issue because of its associations with complications such as jaundice, pancreatitis, and suppurative cholangitis [6]. According to the literature, the risk factors for recurrent CBD stones included in-situ gallbladder with stones, biliary strictures, papillary stenosis, periampullary diverticulum (PAD), and a dilated bile duct without residual obstruction [7].

Recent advances in technology are providing comprehensive knowledge of microbiomes, and it has been well-established that the microbiome of the human gastrointestinal tract plays a critical role in the pathogeneses of many diseases [8]. The roles of microbiomes have been demonstrated in several body sites, including the gastrointestinal tract, oral cavity, nasal cavity, vagina, and skin, using techniques such as next-generation sequencing (NGS) [9]. A previous study reported that the healthy human biliary system is sterile, [10] but Maki et al. suggested that bacteria might play a major role in the pathogenesis of gallstones [11]. Recently, Grigor’eva et al. suggested that bacterial migration to the biliary tract might occur via the sphincter of Oddi, [12] and Ye et al., reported that the biliary tract microbiome is similar to the duodenal microbiome [13]. However, little is known about the relationship between the microbiome and CBD stone recurrence.

In the present study, to determine whether the microbiome influences CBD stone recurrence, we analyzed the biliary microbiome compositions of patients with recurrent CBD stones and compared these of those without recurrent CBD stones, and the biliary and duodenal microbiomes of patients with recurrent CBD stones.

## 2. Methods

### 2.1. Study Design

#### 2.1.1. Bile Microbiome Analysis of Recurrent CBD Stone Patients vs. Non-Recurrent CBD Stone Patients

We conducted bile fluid microbiome analysis on patients that underwent ERCP at our institution from February 2019 to January 2021 to identify differences between the microbiome compositions of SR (recurrent CBD stone) and NSR (non-recurrent CBD stone) patients. Eligible patients were aged 18 years or older, required ERCP for bile duct decompression, and had developed recurrence of CBD stones confirmed by computed tomography (CT) or ultrasound after at least six months of stone removal. Patients with an intrahepatic duct stone, hemolytic anemia, inflammatory bowel disease, or severe liver disease were excluded. Patients with problematic endoscopic approaches due to esophageal stenosis, gastric outlet obstruction, or duodenal stenosis were also excluded, as were patients with a medical history of conditions associated with thrombocytopenia or coagulopathy (PT-INR > 1.5; normal 0.85–1.25, platelet count < 60,000/mm^3^). All data were collected prospectively.

#### 2.1.2. Bile Fluid and Duodenal Microbiomes of Recurrent CBD Stone Patients

Duodenum tissues were obtained from SR patients, recruited as described above, to analyze the microbiome compositions of bile fluid and duodenum tissues. Microbiome compositions were then analyzed to identify differences from the duodenum microbiome composition of controls (gastric ulcer patients without recurrent CBD stones).

#### 2.1.3. Bile Fluid Collection and Duodenal Biopsy under ERCP: Sample Collection

##### Bile Fluid Collection during ERCP

Prior to ERCP, all patients were administrated prophylactic antibiotics (ciprofloxacin 400 mg IV over 60 min) to prevent bacteremia. ERCP was performed using a conventional side-viewing duodenoscope (TJF-260; Olympus Corporation, Tokyo, Japan) and a straight standard injection catheter. After achieving the therapeutic aim of ERCP, an endoscopic nasobiliary drainage (ENBD) tube was inserted such that its proximal end was located in the proximal CBD. Bile samples (20–30 cc) were aspirated 24 h after endoscopic procedures from CBDs via ENBDs to prevent contamination of the upper gastrointestinal tract, including the oral cavity. Samples were immediately placed in germ-free sputum cups and stored at −80 °C until required.

##### Duodenal Tissue Collection during ERCP

Duodenal biopsy was performed prior to biliary access to avoid contamination with duodenal mucosa during ERCP. The duodenum was entered using the conventional side-viewing scope, and two duodenal tissue samples were obtained using biopsy forceps. Duodenal biopsy samples were collected on the opposite side of the ampulla of Vater (AoV) to minimize the risk of bile fluid contamination. Biopsies were performed using disposable biopsy forceps.

##### Duodenal Tissue Collection during Endoscopy for Gastric Ulcer Patients

Esophagogastroduodenoscopy (EGD) procedures were performed using the GIF-H290 duodenoscope (Olympus Co., Ltd., Tokyo, Japan). Two biopsies were obtained from the 2nd portion of the duodenum. Duodenal biopsy specimens were also obtained from the opposite side of the AoV to minimize the risk of bile contamination. Specimens were taken using disposable biopsy forceps.

#### 2.1.4. DNA Extraction from Bile Fluid and Duodenum Tissue

The total bacterial genomic DNA was extracted from 10 mL of bile fluid or duodenal tissue using a Maxwell^®^ RSC PureFood GMO and Authentication Kit (Promega, Madison, WI, USA). Samples were initially centrifuged at 5000× *g* for 5 min at room temperature and then resuspended in 500 µL of cetyltrimethylammonium bromide buffer, according to the manufacturer’s protocol. DNA concentrations were determined using a UV-vis spectrophotometer (NanoDrop 2000c; Thermo Fisher Scientific, Waltham, MA, USA). QuantiFluor^®^ ONE dsDNA System (Promega), and samples were stored at −20 °C until required.

#### 2.1.5. PCR Amplification of the V3–V4 Region of the Bacterial 16S Ribosomal RNA (rRNA) Gene

The V3 and V4 variable regions of the bacterial 16S rRNA gene were amplified using a two-step PCR protocol. Briefly, PCR was conducted using F319 (5′-TCGTCGGCAGCGTCAGATGTGTATAAGAGACAGCCTACG-GGNGGCWGCAG) and R806 (5′-GTCTCGTGGGCTCGGAGATGTGTATAAGAGACA-GGACTACHVGGGTATC- TAATCC-3′) primers. Amplified products were distinguished by 2% agarose gel electrophoresis, and 16S rRNA libraries were purified using AMPure XP magnetic beads, according to the manufacturer’s instructions (Beckman Coulter, Wycombe, UK). A Bioanalyzer 2100 (Agilent, Santa Clara, CA, USA) was used for the sample quality control. For second-round PCR, Illumina Nextera barcodes (Illumina, Inc., San Diego, CA, USA) were attached to first step PCR products using i5 forward primer and i7 reverse primer. Amplified products were purified as described for first-round PCR. DNA quantitation was performed using the QuantiFluor^®^ ONE dsDNA System (Promega). A Bioanalyzer 2100 (Agilent, Santa Clara, CA, USA) was used for sample quality control. A 16S rRNA gene amplification and library preparation (using a two-step PCR protocol) were used to perform 16S rRNA sequencing using a MiSeq v3 Reagent Kit (Illumina, Inc.).

### 2.2. Data Analysis

Sequencing data were processed using the mothur software package (v1.39.4) [14]. DNA sequences were clustered into operational taxonomic units (OTUs) by reference-based OTU clustering using SILVA rRNA database, release 102 [15]. Chimera reads were detected and removed using Chimera UCHIME. Each OTU was assigned taxonomically using the Ribosomal Database Project reference database. Species richness and differences in microbial profiles were demonstrated by alpha and beta diversities calculated using mothur. Microbial diversities, evaluated using OTUs richness, were used to evaluate alpha diversities using Chao1, Shanon, and Simpson indices. Beta diversity refers to compositional dissimilarity between OTUs on phylogenetic trees. To compare group beta diversities, we used non-metric multidimensional scaling (NMDS) and the phyloseq package for R. Linear discriminant analysis with effect size estimation (LEfSe) was conducted to determine differences between relative abundances of taxa between groups. A linear discriminant analysis score > 2 with a *p*-value < 0.05 was considered statistically significant. Data visualization was performed using R (version 3.6.0) and the graphics packages MASS, ggplot2, and reshape2.

### 2.3. Ethics Statement

The study protocol was approved by the Institutional Review Board of Inha University Hospital (INHAUH 2019-02-015) and written informed consents were obtained from all participants before the procedures began. The study was conducted in accordance with the Declaration of Helsinki.

## 3. Results

### 3.1. Characteristics of Patients

Forty-seven patients were enrolled in our study, and their bile fluids were collected and analyzed. Of these, 19 underwent ERCP for recurrent CBD stones (the SR group), and the other 28 patients (the NSR) underwent ERCP for the management of cancer (*n* = 13), benign biliary stricture (*n* = 12), and CBD stones for the first time (*n* = 3). All 28 patients in the NSR group had no prior history of ERCP. Patient baseline characteristics were similar in the two groups. Patient baseline characteristics are summarized in Table 1.

### 3.2. Bile Microbiome Compositions in SR and NSR Groups

**Figure 1:**
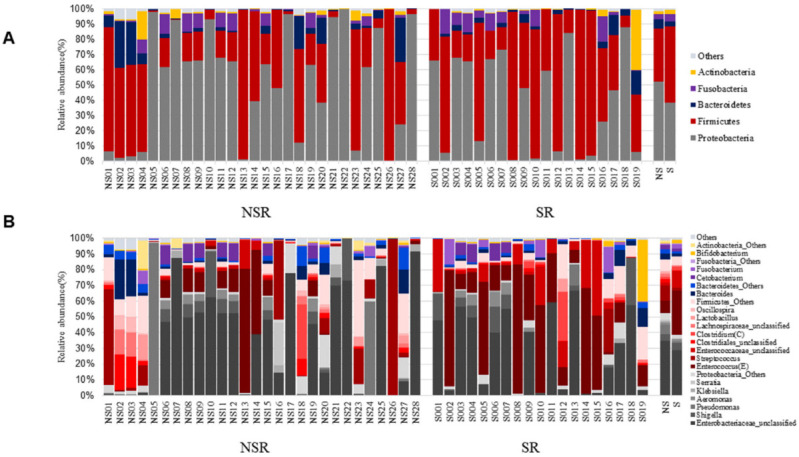
Microbiome compositions at the major phylum and genus levels. (**A**) Phylum level. (**B**) Genus level.

Figure 1 shows microbiome compositions at the major phylum and genus levels in the SR and NSR groups. At the phylum level (Figure 1A), five groups of microbiomes (proteobacteria, firmicutes, bacteroidetes, fusobacteria, actinobacteria) were detected in the bile fluid samples of the 47 patients at an average percentage composition of ≥ 1% of total microbiomes. In both groups, proteobacteria and firmicutes accounted for most of the microbiomes. In the SR group, fusobacteria, bacteroidetes, and actinobacteria were detected in decreasing order of abundance, while in the NSR group, bacteroidetes, fusobacteria, and actinobacteria were detected in of abundance. Phyla that accounted for <1% of the total microbiome were designated as ‘others’. At the genus level of the proteobacteria phylum, enterobacteriaceae_unclassified was the most common in both groups. At the genus level, firmicutes phylum, enterococcus, and enterococcaceae_unclassified were most abundant in the SR group, whereas in the NSR group, streptococcus and enterococcus were most abundant (all in decreasing order) (Figure 1B). As was performed at the phylum level, genera occupying <1% were collected and expressed as ‘others’.

### 3.3. Microbial Community Heterogeneities in the SR and Control Groups

**Figure 2:**
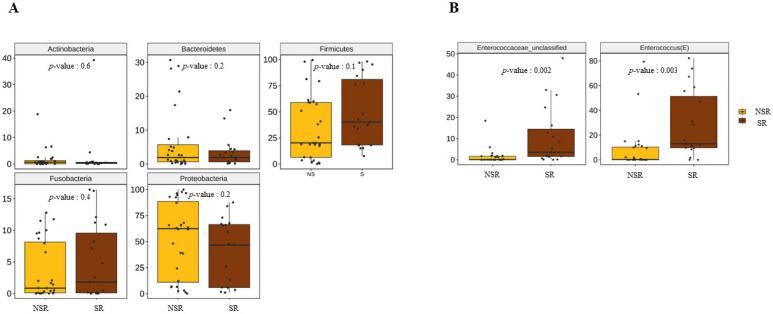
Taxonomic compositions at the major phylum and genus levels. (**A**) Phylum level. (**B**) Genus level.

The significances of taxonomic compositional differences were determined using the ANOVA range test and Turkey’s HSD test. At the phylum level, no significant difference was observed between the compositions of microorganisms in the SR and NSR groups (Figure 2A). However, enterococcus and enterococcaceae_unclassified were significantly more abundant at the genus level in SR group. (Figure 2B)

**Figure 3:**
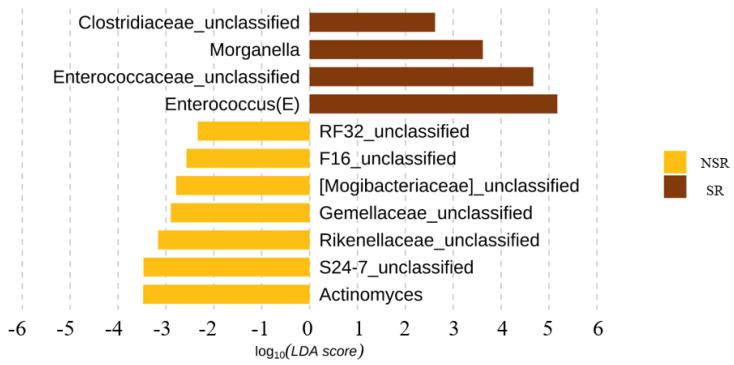
Linear discriminant analysis with effect size (LEfSe) at the genus level.

**Figure 4:**
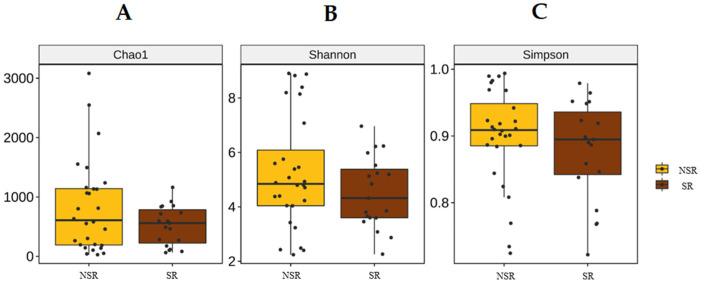
Alpha diversity. (**A**) Chao1. (**B**) Shannon. (**C**) Simpson.

**Figure 5:**
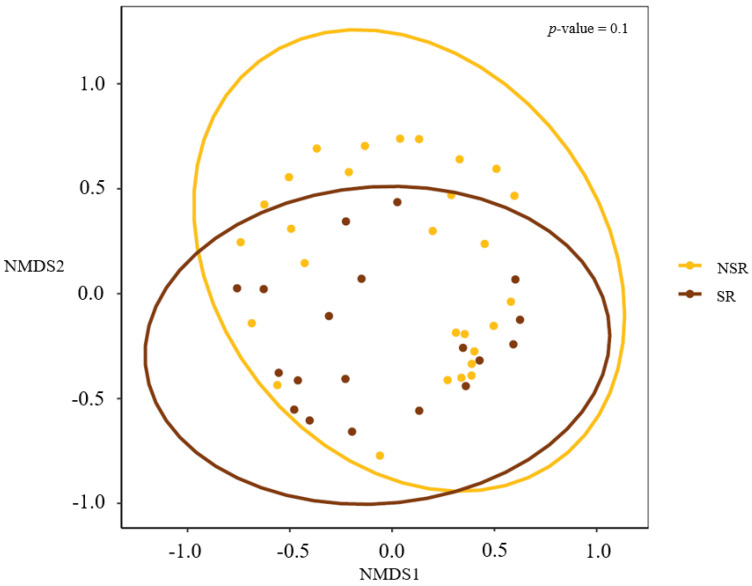
Beta diversity. Non-metric Multidimensional Scaling (NMDS) ordination of beta diversity analyses at the ASV (Amplicon Sequence Variant) level did not exhibit any significant differences between the SR and NSR groups (*p* = 0.1).

Linear discriminant analysis effect sizes (LEfSe) were determined to identify major differences between the biliary microbiomes of the SR and NSR groups. Based on linear discriminant analysis (LDA) selection, enterococcus (LDA score 5.2) and enterococcaceae_unclassified (LDA score 4.7) proportions were significantly greater in the SR group at the genus level. (Figure 3) Alpha diversity metrics were assessed to quantify heterogeneities of microbial communities in samples. A comparison of alpha diversity scores of the SR and NSR groups using the ANOVA range test and Turkey’s HSD test failed to reveal any significant difference (Figure 4). We attributed this null result to compromised patient health due to the presence of pancreatic or biliary diseases. AMOVA testing and Non-metric Multidimensional Scaling (NMDS) ordination of beta-diversity analyses at the ASV (Amplicon Sequence Variant) level showed no significant differences between the SR and NSR groups (*p* = 0.1) (Figure 5).

### 3.4. Relationships between the Bile and Duodenal Microbiome and CBD Stone Recurrence

Several studies have reported that recurrent CBD stone is related to duodenal biliary reflux. To analyze the microbiomes of duodenal tissues and elucidate the origin of bile enterococcus in the SR group, we analyzed duodenal mucosa biopsy and bile fluid samples obtained from five patients in the SR group. The baseline characteristics of the patients are summarized in Supplementary Table S1.

**Figure 6:**
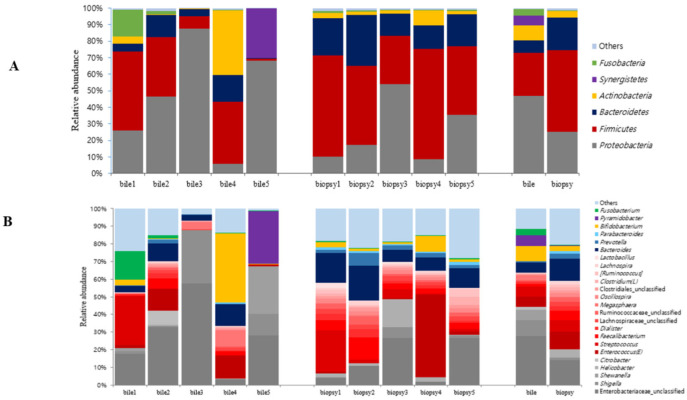
Microbiome compositions of duodenum tissue and bile fluid samples of five recurrent CBD stone patients. (**A**) Phylum level. (**B**) Genus level.

A comparison of the microbiome compositions of duodenum and bile fluid of these five patients showed that for each patient, proteobacteria compositions were almost identical in bile fluid and duodenal mucosa from the genus to phylum levels (Figure 6). Furthermore, proteobacteria and firmicutes largely accounted for compositions at the phylum level in bile fluid and duodenal mucosa (Figure 6A). At the genus level analysis, the most common genera in bile fluid and duodenum tissues were enterobacteriaceae_unclassified in the proteobacteria phylum and enterococcus in the firmicutes phylum (Figure 6B).

**Figure 7:**
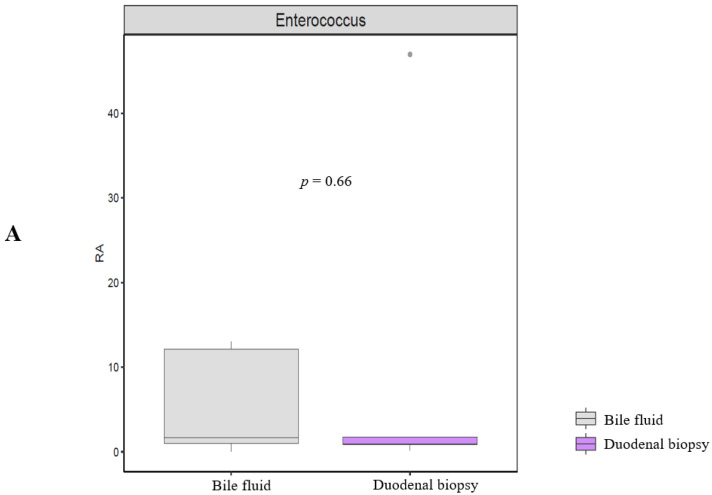

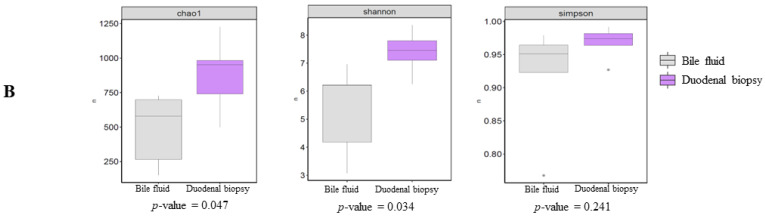
Taxonomic enterococcus composition and alpha diversity analysis. (**A**) Taxonomic enterococcus composition. (**B**) Alpha diversity (Chao1, Shannon, and Simpson indices).

Taxonomic compositions were analyzed using the ANOVA range test and Turkey’s HSD test at the genus level. No significant (*p* = 0.66, Welch’s *t*-test) enterococcus compositional difference was observed between bile fluid and duodenal mucosa at the genus level. (Figure 7A).

**Figure 8:**
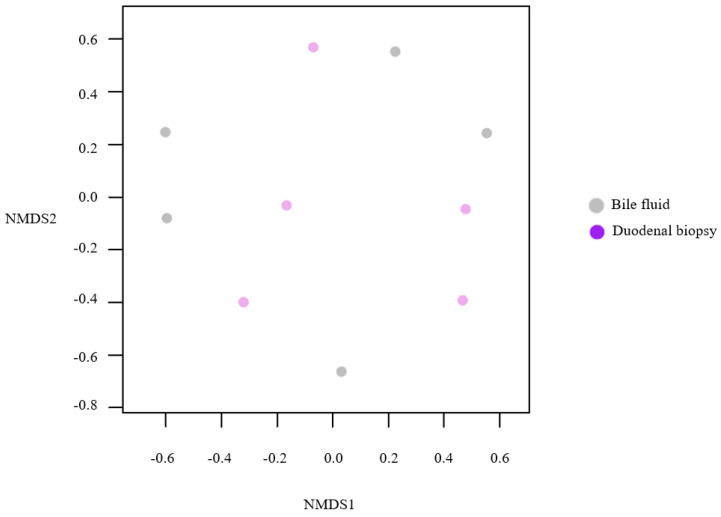
Beta diversity (NMDS analysis of microbial OTU levels).

Additionally, alpha-diversities of bile fluid and duodenal mucosa were determined using Chao1, Shannon and Simpson diversity indices. (Figure 7B) The microbiome compositions of the bile fluid and duodenal mucosal samples collected from the five SR patients were analyzed, and Chao1 and Shannon indices were significantly different, but Simpson indices were not. (Chao1: *p* = 0.047; Shannon: *p* = 0.034; Simpson: *p* = 0.241) These results indicate that microbiome compositions and environments are similar in the bile duct and duodenum in recurrent CBD stone patients. Subsequently, we compared enterococcus compositions in bile fluid and duodenum, which differed in the SR group, in order to investigate the possibility that enterococcus in bile fluid originated from duodenum. We conducted NMDS ordination based on Bray–Curtis’s dissimilarities in genetic diversity in a microbial community to cluster 16S rRNA sequences into OTUs based on sequence similarities (Figure 8). NMDS analysis showed no significant difference between the microbial environments of bile fluid and duodenal biopsy samples in the five SR patients (*p* = 0.46). These results supported the notion that enterococcus in bile fluid originated from duodenum.

### 3.5. Microbial Community Heterogeneity between the Bile and Duodenal Microbiomes of Recurrent CBD Stone Patients and the Duodenal Microbiome of Controls

Finally, to compare the microbiome compositions of duodenal mucosae in the SR group with controls (gastric ulcer patients, *n* = 12) without any kind of biliary disease, duodenal mucosa biopsy samples were collected and analyzed. The baseline characteristics of controls are summarized in Supplementary Table S1.

**Figure 9:**
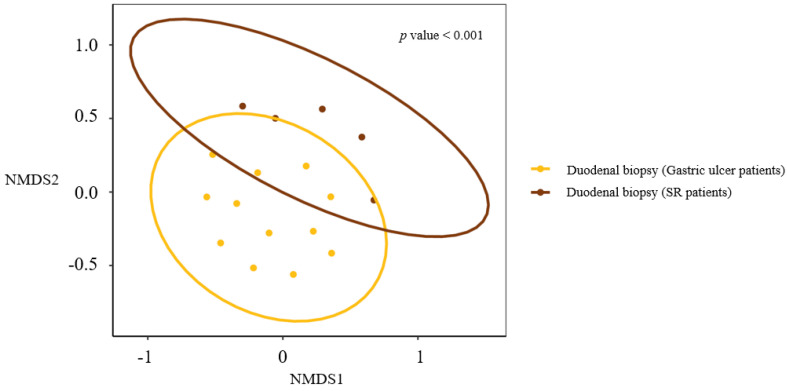
NMDS ordination of beta-diversity analyses at the ASV level between the SR and NSR groups.

NMDS ordination of beta-diversity analyses at the ASV level demonstrated a significant difference between the duodenal compositions of the SR group and controls, indicating the environments represented differed. The beta diversity analysis of microbiome diversity based on weighted Braycurtis-distance also showed the duodenal microbial environments in SR patients and controls to be significantly different. (Figure 9) Supplementary Figure S1. illustrates NMDS ordination of beta-diversity analyses at the ASV level between the SR group (duodenum tissues and bile fluid) and controls (duodenum tissues).

## 4. Discussion

In the present study, we found that enterococcaceae_unclassified and enterococcus were significantly more abundant in the bile fluid samples of the SR group than in those of the NSR group (*p* = 0.002 and *p* = 0.003). Verification using the LEfSe test showed that LDA scores were 5.2 and 4.7 for enterococcus and enterococcaceae_unclassified, respectively. In addition, the microbiome compositions and beta diversities of bile fluid and duodenal tissues in the SR group were similar (*p* = 0.46).

According to a recent study on the microbiome in patients with recurrent CBD stones, microbiome dysbiosis affects the rate of CBD stone recurrence. In addition, seven genera, including enterococcus, were found more frequently (albeit non-significantly) in patients with stone recurrence [16]. In our study, significant biliary microbial alterations were observed in the SR group as compared with the NSR group. More specifically, enterococcus, enterococcaceae_unclassified, morganella, and clostridiaceae_unclassified were more abundant in the SR group (in the LEfSe of the genus level), and enterococcus was significantly more abundant. In a previous study, we showed that enterococcus might play an important role in the development of choledocholithiasis, and the current study indicates that it might play an important role in CBD stone recurrence [17]. Enterococcus is a large genus of lactic acid bacteria of the phylum Firmicutes, and this genus is tolerant of a range of environmental conditions, including a wide range of pH values (4.5–10.0) and high sodium chloride concentrations, [18] which suggests that enterococcus might have the potential to overcome the bacteriostatic and bactericidal environment of bile acids [19].

The bile microbiome has a unique composition quite different from that of the duodenum. Recent studies have shown that the composition of the bile duct microbiome differs from those of upper digestive sites such as the oral cavity and duodenum [20]. Furthermore, several authors have suggested that retrograde infection from the duodenal microbiome could be the source of biliary infections [16,21,22]. In a recent study, Han et al. reported similarities between the microbiomes of duodenal juice and bile fluid in patients with choledocholithiasis and suggested the possibility of an association between duodenal-biliary reflux and choledocholithiasis at the microbiome level [22]. However, they did not show that the compositions of patients’ samples were similar. On the other hand, the present study shows that the microbiome compositions of bile fluid and duodenal mucosa were similar in recurrent CBD stone patients. Furthermore, Han et al. excluded recurrent stones, and thus, their patient population differed from that of our study. In addition, only 10 samples were analyzed, and no comparisons with controls were attempted. To the best of our knowledge, the current study is the first to report microbial similarities between duodenum tissues and bile fluid in patients with recurrent CBD stones at the microbiome level. Furthermore, these similarities strongly support the hypothesis that microbiome migration from the duodenum to the biliary system might underlie the pathogenesis of CBD stone recurrence.

Interestingly, the microbial composition of the duodenum in the SR group was significantly different from that in the NSR group, but beta diversities of bile and duodenal microbiomes were similar in the ASV level in the SR group (Figure 9). These findings encourage us to hypothesize that retrograde migration of the duodenal microbiome may alter the biliary microbiome and contribute to the pathogenesis of CBD stone formation.

The present study has a number of limitations that warrant consideration. First, due to ethical problems, we were unable to enroll healthy normal controls, and thus, were unable to obtain bile or duodenal samples from healthy individuals. Second, we used bile fluid and duodenal biopsy for the analysis, and bile duct biopsies may have more accurately revealed microbial communities in the biliary tract, as it is possible that bacteria species in bile fluid did not reflect the microbial environments of biliary tracts. Third, our study was conducted at a single center on Korean patients, and thus, caution should be exercised when generalizing our results to other races and diet patterns. Fourth, information obtained by 16S rRNA gene sequencing is not as reliable as that obtained by shotgun metagenomic sequencing of entire DNA. Fourth, our study did not investigate the mechanism by which enterococcus affects the formation of recurrent CBD stones. Further studies are needed to clarify the relevant mechanisms.

## 5. Conclusions

In conclusion, the present study demonstrates that enterococcus was significantly more abundant in the bile fluid of patients with recurrent CBD stones than in patients without, and no significant difference was observed between the microbiome compositions of bile fluid and duodenal tissue in recurrent CBD stone patients. This observed similarity between the biliary and duodenal microbiome supports the notion that the duodenal microbiome plays an important role in CBD stone recurrence.

## Figures and Tables

**Table 1:** Patient Baseline Characteristics of SR and NSR groups.

Variables	Total (*n* = 47)	SR (*n* = 19)	NSR (*n* = 28)	*p* *
Age (years) ^§^	75 (42–99)	78 (42–99)	75 (42–88)	0.44
Sex, male, *n* (%)	25 (53.2%)	7 (36.8%)	18 (64.3%)	0.07
HTN, presence, *n* (%)	21 (44.7%)	8 (42.1%)	13 (61.9%)	0.78
DM, presence, *n* (%)	12 (25.5%)	6 (31.6%)	6 (21.4%)	0.44
Dyslipidemia, presence, *n* (%)	19 (40.4%)	8 (42.1%)	11 (39.3%)	0.85
WBC (/uL)	8779.4 (3050–29,130)	8152.6 (3710–18,480)	9204.6 (3050–29,130)	0.51
CRP (mg/dL)	5.1 (0.1–28.7)	7.0 (0.1–24.0)	3.7 (0.1–28.7)	0.13
Total bilirubin (mg/dL)	4.4 (0.2–27.9)	2.5 (0.3–10.4)	5.6 (0.2–27.9)	0.09
AST (IU/L)	154.9 (12.0–663.0)	151.2 (12.0–535.0)	157.4 (19.0–663.0)	0.91
ALT (IU/L)	136.5 (8.0–688.0)	139.4 (8.0–510.0)	134.5 (10.0–688.0)	0.92
ALP (IU/L)	368.6 (41.0–3435.0)	135.7 (41.0–267.0)	526.7 (45.0–3435.0)	0.05

Abbreviation: HTN, hypertension; DM, diabetes mellitus; WBC, white blood cell count; CRP, c-reactive protein; AST, alanine aspartatetransferase; ALT, alanine aminontransferase; ALP, alkaline phosphatase. ^§^, median (range); *, *p* values were calculated using the *t*-test or the *Chi*-square test.

## Data Availability

The raw data are available from the corresponding author on reasonable request, and planning to be shared in the public repository, NCBI database. Data sharing is not applicable to this article as no datasets were generated or analysed during the current study.

## Supplementary Materials

The following supporting information can be downloaded at: https://www.mdpi.com/article/10.3390/life12101540/s1, Table S1: Baseline Characteristics of the five recurrent CBD stone patients and the 12 gastric ulcer patients, Figure S1: NMDS ordination of beta-diversity analyses at the ASV level between the SR group (duodenum tissues and bile fluid) and controls (duodenum tissues).

## Abbrevations

CBD	common bile duct
ERCP	endoscopic retrograde cholangiopancreatography
EST	endoscopic sphincterotomy
PAD	periampullary diverticulum
NGS	next-generation sequencing
ENBD	endoscopic nasobiliary drainage
AoV	ampulla of Vater
EGD	Esophagogastroduodenoscopy
