# Supplementary material for: Bile Microbiome in Patients with Recurrent Common Bile Duct Stones and Correlation with the Duodenal Microbiome
Source: Life (Basel). 2022 Oct 3;12(10):1540. doi: 10.3390/life12101540 (PMC9605223; doi:10.3390/life12101540)
Supplement: Supplementary file 1 [file life-12-01540-s001.zip › supplementary .pdf]

Supplementary

Table S1. Baseline Characteristics of the five recurrent CBD stone patients and the 12 gastric ulcer patients.

**Variables**	**Recurrent CBD stone patient (n=5)**	**Gastric ulcer patients (n=12)**
Age (years) §	78 (56-99)	69.5 (43-85)
Sex, male, n (%)	40.0%	58.3%
HTN, presence, n (%)	40.0%	41.7%
DM, presence, n (%)	80.0%	33.3%
Dyslipidemia, presence, n (%)	40.0%	8.3%
WBC (/ul)	7,000.0 (4,730.0-10,060.0)	5,370.8 (3,240.0-8,840.0)
CRP (mg/dL)	5.0 (0.1-11.0)	0.6 (0.0-1.9)
Total bilirubin (mg/dL)	2.7 (0.6-10.4)	0.5 (0.2-1.0)
AST (IU/L)	132.2 (15.0-277.0)	19.3 (12.0-37.0)
ALT (IU/L)	148.6 (9.0-356.0)	14.4 (6.0-20.0)
ALP (IU/L)	160.4 (95.0-231.0)	74.3 (44.0-140.0)

Abbreviation: HTN, hypertension; DM, diabetes mellitus; WBC, white blood cell count; CRP, c-reactive protein; AST, alanine aspartatetransferase; ALT, alanine aminontransferase; ALP, alkaline phosphatase

§, median (range)

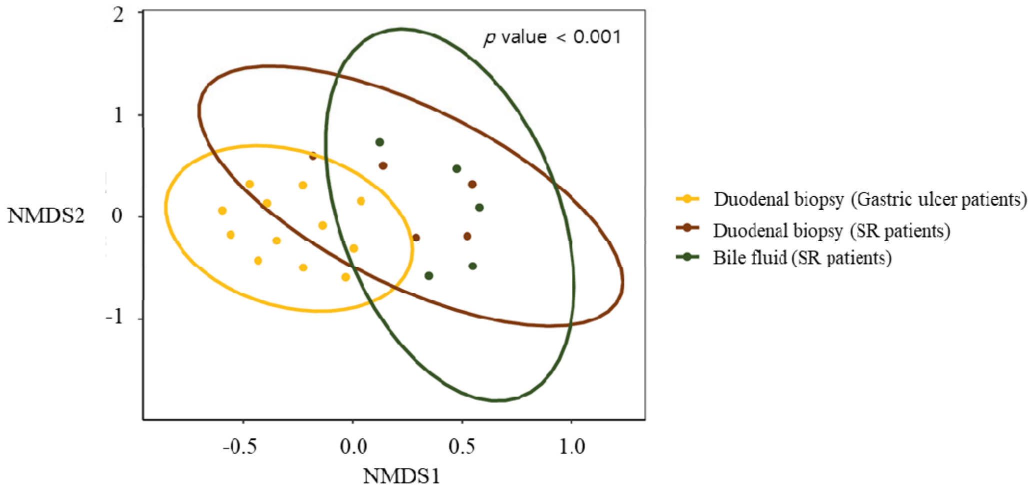

Figure S1. NMDS ordination of beta-diversity analyses at the ASV level between the SR group (duodenum tissues and bile fluid) and controls (duodenum tissues).
